# Antimicrobial Effect of Graphene in Dentistry: A Scoping Review

**DOI:** 10.3390/dj13080355

**Published:** 2025-08-05

**Authors:** Ricardo Martuci, Susana João Oliveira, Mateus Martuci, José Reis-Campos, Maria Helena Figueiral

**Affiliations:** 1 Faculdade de Medicina Dentária, Universidade do Porto, Rua Dr. Manuel Pereira da Silva, 4200-393 Porto, Portugal; rmartuci@fmd.up.pt (R.M.); jcampos@fmd.up.pt (J.R.-C.); 2 Instituto de Engenharia Mecânica, Universidade Federal de Itajubá, Itajubá 37500-903, Brazil; d2020020891@unifei.edu.br; 3 Instituto de Ciência e Inovação em Engenharia Mecânica e Engenharia Industrial (INEGI), Universidade do Porto, Campus da FEUP, R. Dr. Roberto Frias 400, 4200-465 Porto, Portugal

**Keywords:** graphene, graphene oxide, antimicrobial effect, bactericidal effect, dental biomaterials

## Abstract

**Background/Objectives**: The functionalization of various forms of graphene, such as graphene nanoplatelets, graphene oxide, and reduced graphene oxide, in biomaterials is a promising strategy in dentistry, particularly regarding their antimicrobial potential. However, conclusive studies on the toxicity and biocompatibility of graphene-based materials remain limited, and standardized guidelines for their production, handling, and dental applications are still lacking. This scoping review aims to map the available studies on various types of graphene, synthesize evidence on their antimicrobial effectiveness, and describe the main biological responses when functionalized in dental biomaterials. **Methods**: An electronic search was conducted in the Clarivate, PubMed, and Scopus databases using the descriptors as follows: ‘graphene’ AND ‘antimicrobial effect’ AND ‘bactericidal effect’ AND (‘graphene oxide’ OR ‘dental biofilm’ OR ‘antibacterial properties’ OR ‘dental materials’). Article screening and eligibility assessment were performed based on predefined inclusion and exclusion criteria, following the PRISMA-ScR guidelines. **Results**: The search identified 793 articles. After removing duplicates, applying the eligibility criteria, and performing a full-text analysis of 64 articles, 21 studies were included in the review. Graphene oxide, particularly at low concentrations, was the most commonly studied graphene variant, demonstrating significant antimicrobial efficacy against *S. mutans*, *S. faecalis*, *E. coli*, *P. aeruginosa*, and *C. albicans*. Both mechanical and chemical mechanisms have been linked to the biological responses of graphene-doped biomaterials. The biocompatibility and cytotoxicity of these compounds remain controversial, with some studies reporting favorable outcomes, while others raise significant concerns. **Conclusions**: Graphene shows great promise as an antimicrobial agent in dental biomaterials. Despite encouraging results, more in vitro and in vivo studies are needed to better understand its biocompatibility and cytotoxicity in dental applications. Additionally, standardized production protocols, clearly defined clinical applications in dentistry, and regulatory guidelines from the World Health Organization concerning handling procedures and occupational risks remain necessary.

## 1. Introduction

The continuous emergence of drug-resistant microorganisms is well recognized in dentistry, primarily due to the overuse or misuse of antibiotics in a variety of dental procedures, including periodontal therapy, endodontics, and implant-related treatments [1]. The discovery of alternative antimicrobial agents is, therefore, of utmost importance and has increased the attention toward graphene-based materials.

Graphene is a two-dimensional structure composed of carbon atoms arranged in a hexagonal lattice. It has received increasing attention since the 1950s, when Linus Pauling addressed its potential applications in molecular biology and medicine. The interest in this material evolved over the years, with Philip Russell Wallace thoroughly exploring its properties.

The term ‘graphene’ was first introduced in 1987, but it was only in 1994 that the International Union of Pure and Applied Chemistry (IUPAC) officially recognized it. In 2004, researchers Andre Geim and Kostya Novoselov from the University of Manchester successfully isolated graphene through chemical exfoliation of bulk graphite, an achievement that was honored with the Nobel Prize in Physics in 2010 [2,3]. Since then, novel methods for the fabrication of 3D graphene structures have been developed, among which the laser-induced graphene (LIG)—first produced on polyimide substrates through laser ablation—has attracted particular attention in recent years due to its wide range of applications, accuracy, cost-effectiveness, and energy efficiency [4].

Due to their unique mechanical, optical, chemical, and electrical properties, graphene-based biomaterials are considered promising building blocks for numerous biological and biomedical applications, including drug and molecule delivery systems, bioimaging, photothermal therapy, biosensing, tissue engineering, and regenerative medicine [5]. In the field of dental medicine, potential applications of graphene are continuously expanding, ranging from bone tissue engineering and regeneration to scaffold materials for osteogenic, osteoblastic, and cementoblast differentiation, as well as implant coatings, restorative dentistry, and additives in endodontic irrigants and cements [6].

**Figure 1:**
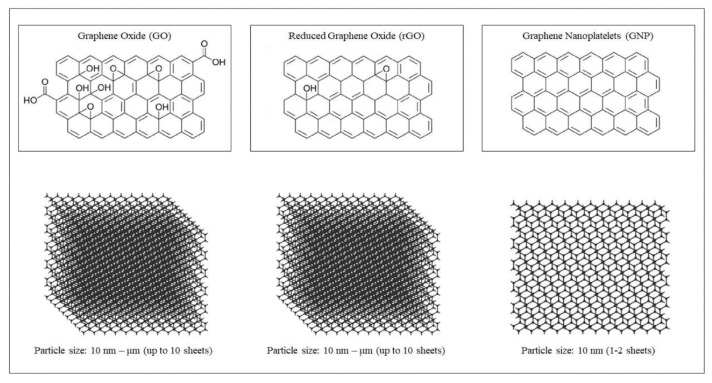
Depiction of the various types of graphene. Reproduced from Ref. [29].

Primarily recognized for its mechanical properties in dentistry, various types of graphene (Figure 1)—encompassing graphene nanoplatelets (GNP), graphene oxide (GO), reduced graphene oxide (rGO), and nitrogen-doped graphene—also exhibit notable antimicrobial activity. This antimicrobial effect has been associated with bacterial death resulting from the rupture of the cell membrane upon contact with the graphene nanosheet interface. The nanosheets’ sharp edges (often referred to as nanoknives) and dimensions physically disrupt the membrane, leading to cell death. This mechanism, known as mechano-bactericidal [7], arises from the modified nanotopography of the biomaterial surface, which creates a physically hostile environment that eliminates bacteria through biomechanical damage [8]. Additional antimicrobial mechanisms have been reported in the literature, wherein bacterial cell death is induced by the intrinsic chemical and physical properties of graphene. Its nanoscale bactericidal effect is attributed to several mechanisms, including biochemical interactions, oxidative stress, exposure to oxidizing agents, ionic release, protein denaturation, chemical antagonism, electrical conductivity [9], photothermal irradiation [10], combination with bioactive molecules, and hydrophilicity [11].

Studies on graphene-functionalized biomaterials, with varying interface exposure periods, have demonstrated favorable antimicrobial results and confirmed their effectiveness in the complete elimination of *Staphylococcus aureus*, *Staphylococcus epidermidis*, *Streptococcus mutans*, *Streptococcus pyogenes*, *Klebsiella pneumoniae*, *Bacillus cereus*, *Porphyromonas gingivalis*, *Fusobacterium nucleatum*, *Escherichia coli*, *Bacillus subtilis*, *Pseudomonas aeruginosa*, *Pseudomonas putida*, *Salmonella enterica*, *Salmonella typhimurium*, *Salmonella choleraesuis*, *Enterococcus faecalis*, *Caenorhabditis elegans*, *Serratia marcescens*, *Helicobacter pylori*, and members of the Enterobacteriaceae family, among other Gram + and Gram − bacteria, as well as the fungi *Candida albicans* and *Rhizopus oryzae* [12,13,14,15,16,17,18]. 

While the significance of graphene-based composites and their antimicrobial mechanism has been addressed in different reviews [19,20], conclusive studies on their toxicity and biocompatibility remain limited. These parameters are critical factors that complement graphene’s antimicrobial effect. The possible adverse, toxic, or carcinogenic reactions associated with the clinical application of graphene are still poorly understood. Although some in vitro and in vivo studies have reported environmental and health hazards in humans, generalizations regarding the toxicity of graphene-based materials should be made with caution, as these effects are highly dependent on graphene’s type and specific application [21]. In an in vivo study using mice, oral administration of GO nanoparticles showed that graphene can be toxic to living tissues. The study also reported the degradation of graphene sheets by macrophages and neutrophils, attributed to the phagocytic activity of these cells [22,23]. Investigations into the various forms of graphene, correlating their toxicity with living tissues, have yielded inconsistent results. GO demonstrated the lowest toxicity and excellent biocompatibility, likely due to its antioxidant properties [24,25].

Additional gaps identified in the literature include the absence of standardized dental protocols for the handling and application of graphene-based materials, occupational safety concerns related to working with these nanomaterials [26,27], and the lack of regulatory guidelines from the World Health Organization (WHO), all of which collectively hinder the widespread use of graphene in dentistry. In light of these gaps, a comprehensive and updated analysis of the available data—particularly regarding occupational risks, dose–response toxicity, and tissue regeneration—is essential for establishing new antimicrobial guidelines that will support the safe integration of graphene into dental practices [28].

This scoping review aims to map the available studies on various types of graphene, synthesize evidence on their antimicrobial effectiveness, and describe the main biological responses when functionalized in dental biomaterials.

## 2. Materials and Methods

The databases consulted were Clarivate, PubMed, and Scopus, selected for their comprehensiveness and reliability. The descriptors used were ‘graphene’ AND ‘antimicrobial effect’ AND ‘bactericidal effect’ AND (‘graphene oxide’ OR ‘dental biofilm’ OR ‘antibacterial properties’ OR ‘dental materials’). The electronic research was conducted using all fields between September and December 2024.

This scoping review followed the recommendations outlined in the PRISMA-ScR guidelines [30,31], but it was not registered in PROSPERO. The PICO strategy (Table 1) was employed to formulate the research question guiding the study: “What are the antimicrobial effects of graphene, regardless of its type, in dental biomaterials?”. The inclusion and exclusion criteria are listed in Table 2.

To create the database, studies were selected after a thorough screening of the abstracts. Subsequently, the full texts of the selected articles were read for review. The reference database was analyzed and managed using EndNote (version 21.0, Clarivate, Philadelphia, PA, USA). The screening process was independently performed by two reviewers, and any disagreements were resolved through discussion or consultation with a third reviewer. The data analysis was conducted as follows: the full texts of the articles included in the study were read, and the information was transcribed into a spreadsheet using the sequence author, publication year, objective, broad-spectrum antimicrobial activity, types of graphene, target microorganisms, biological responses of graphene, antibiotic resistance, and conclusions, in accordance with Table 3.

## 3. Results

**Figure 2:**
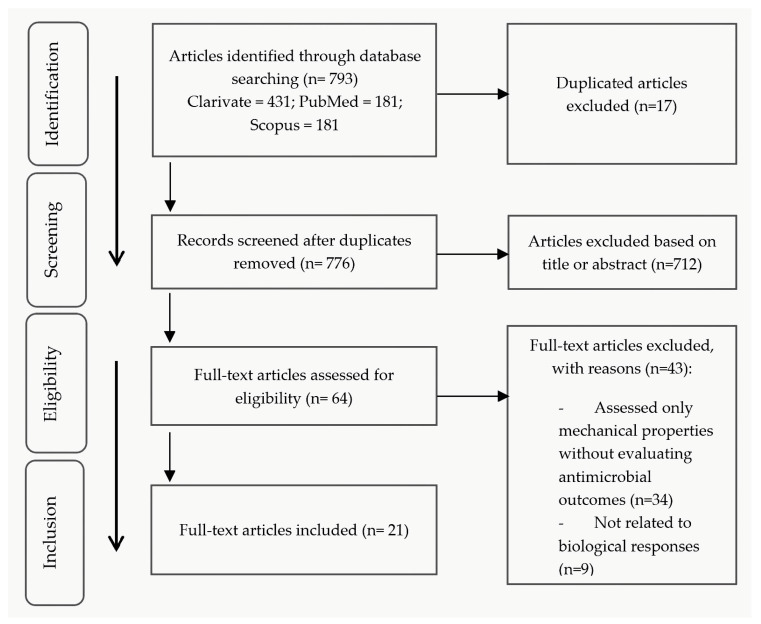
PRISMA flow diagram used in the scoping review process.

The search identified 793 articles, and after removing duplicates, 776 articles were assessed. Based on the title and abstract, 712 articles were excluded. After applying the eligibility criteria, 64 full texts were analyzed, and upon review, 21 articles were included in the study (Figure 2). 

GO was the most commonly studied form of graphene in the included studies. Its antimicrobial effect, particularly when incorporated into dental biomaterials at low concentrations, demonstrated significant broad-spectrum antimicrobial efficacy against microorganisms such as *S. mutans*, *S. faecalis*, *E. coli*, *P. aeruginosa*, and *C. albicans* after 24 h of exposure, while maintaining favorable mechanical properties [8,9,13,18,32]. Mechano-bactericidal mechanisms—associated with the morphology of graphene nanosheets and nano-wrapping phenomena—have been reported to underlie the antimicrobial properties of graphene. However, mechanical parameters such as morphology, size, solubility, dispersibility, and hydrophilicity of graphene sheets require further investigation to fully elucidate their role in mechano-bactericidal effectiveness [7,12,24,33]. Additionally, chemical and physical parameters, including the control of oxidative stress and surface electrical conductivity, directly influence the biological responses to graphene, particularly its biocompatibility, cytotoxicity, and potential for tissue regeneration [10,14,27].

The diversity of research designs to assess the antimicrobial response of graphene-based biomaterials is noteworthy, which often limits cross-study comparisons. Among the included studies, the most commonly used protocols were broth dilution methods [7,8,11,18], disk diffusion assays [13,26], and the direct application of the biomaterial as a substrate [15,16,17,22,25,26,34,35,36].

The biocompatibility and cytotoxicity of graphene-based materials were not consistently addressed across all included studies. While several reports demonstrated favorable biocompatibility [8,13,15,16,17,18,24,25,27,32,33], others emphasized the need for further in vitro and in vivo investigations [12,34]. The cytotoxic potential of graphene-doped materials remains controversial, with some studies raising significant concerns [9,12,13,16,17,24,27,34].

The conjugation of GO with other biomaterials and biodegradable polymeric matrices was explored in several of the selected studies, including combinations with polymethylmethacrylate (PMMA) [26,32], polyetheretherketone (PEEK) [35,36], and chitosan [18]. Overall, such functionalization enhanced the antimicrobial effect and bioactivity of the resulting biomaterials while preserving or even improving their mechanical properties.

Table 3 summarizes the key findings on the biological response to graphene as outlined in the articles included in this review, arranged in chronological order to provide insight into the evolution of studies.

**Table 3:** Distribution of selected studies based on the biological responses of graphene, arranged in chronological order.

Authors and Year	Objectives	Broad-Spectrum Antimicrobial Activity * of Graphene			Types of Graphene ^#^	Target Microorganisms (Cell Inactivation and/or Lysis)	Biological Responses of Graphene ^‡^				Conclusions
		Mechanical Mechanisms	Physical or Chemical Mechanisms	Antibiotic-Resistant Human Infections			Biocompatibility	Cytotoxicity	Tissue Regeneration	Occupational Risk	
**Akhavan et al.** 2011 **[7]**	Inactivation of bacterial bioactivity	Morphology of graphene nanosheets Nano-wrapping	Oxidative stress Photothermal irradiation		GNP (2 g/50 mL)	*E. coli*					Combining graphene with infrared irradiation enhances its broad-spectrum antimicrobial effect
**Bressan et al.** 2014 **[27]**	Surface chemical modification by graphene		Oxidative stress Photothermal irradiation Electrical conductivity		GO		Observed (oxidative stress control)	Observed (oxidative stress control)	Observed	Inhalation risk	Graphene stimulates bone cell differentiation
**Zanni et al.** 2016 **[8]**	Research on biomaterials with graphene	Morphology of graphene nanosheets Nano-wrapping	Oxidative stress Photothermal irradiation Electrical conductivity		GNP (50 µg/mL)	*S. mutans* *P. aeruginosa* *C. elegans*	Observed		Observed		Graphene showed efficacy in controlling *S. mutans*
**Guazzo et al.** 2018 **[24]**	Applications for graphene-functionalized materials	Morphology of graphene nanosheets Nano-wrapping	Photothermal irradiation		GO rGO		Observed	Observed	Observed		The behavior of the biomaterial depends on the properties of graphene
**Linklater et al.** 2018 **[9]**	Review the bactericidal effect of graphene and graphene-derived materials		Photothermal irradiation Electrical conductivity		GO	*E. coli* *P. aeruginosa* *B. subtilis*		Observed		No protocols Dangerous use	The chemical surface effects of graphene-induced bacterial cell membrane rupture
**Azevedo et al.** 2019 **[25]**	Assess the biological response of soft tissues	Morphology of graphene nanosheets Nano-wrapping	Oxidative stress Photothermal irradiation Electrical conductivity		GO + PMMA		Observed				Biological benefits for health
**Ghorbanzadeh et al.** 2020 **[10]**	Evaluate the potential for E. faecalis inhibition in endodontic therapy	Morphology of graphene nanosheets	Oxidative stress Photothermal irradiation		rGO (250 µg/mL) (125 µg/mL)	*E. faecalis*					Positive effect for endodontic infection control
**Nichols F & Chan S** 2020 **[33]**	Alternative antibiotic agents	Morphology of graphene nanosheets Nano-wrapping	Oxidative stress Photothermal irradiation	Correlation considered	GO rGO	*E. coli* *S. epidermidis*	Observed (oxidative stress control)				Development of high-performance antimicrobial agents
**Zhao et al.** 2020 **[11]**	To determine the optimal concentration of GO	Morphology of graphene nanosheets Nano-wrapping	Oxidative stress		GO (40 µg/mL) (80 µg/mL)	*S. mutans*					Improved results from the association of nanosheets with oxidative stress
**Alavi et al.** 2021 **[16]**	Evaluation of bactericidal effect and bio compatibility	Morphology of graphene nanosheets Nano-wrapping	Photothermal irradiation Electrical conductivity	Correlation considered	GO	Gram + and Gram −	Observed	Concerns with the optimal dose of graphene			Introducing other types of carbon derivatives does not increase the graphene content
**Avcu et al.** 2022 **[18]**	Evaluation of polymer matrix composites containing graphene	Morphology of graphene nanosheets Nano-wrapping	Oxidative stress Photothermal irradiation Electrical conductivity		GO (1–6 wt%) rGO GNP (better results with 0.5 wt%)	More than 30 types of bacteria including *H. pylori and S. pyogenes*	Observed (increases with the presence of chitosan)	Decreases due to chitosan (biodegradation)			The combination of graphene and chitosan enhances the antimicrobial effect
**Salgado et al.** 2022 **[26]**	Evaluation of antimicrobial effect of 3D PMMA	Morphology of graphene nanosheets Nano-wrapping				*S. mutans* *C. albicans*					The presence of graphene is effective in inhibiting microbial growth
**Zhou et al.** 2022 **[19]**	Evaluation of antimicrobial effect of hybrid biomaterials	Morphology of graphene nanosheets Nano-wrapping			GO rGO					No risk	Graphene materials do not exhibit bacterial resistance
**Butler et al.** 2023 **[13]**	Development of medical and dental antimicrobial biomaterials	Morphology of graphene nanosheets Nano-wrapping	Oxidative stress Electrical conductivity	Correlation considered	GNP GO rGO	Various Gram + and Gram − bacteria Fungus *Rhizopus oryzae*	Observed (increases with the presence of chitosan and silica)	Toxic potential and ecological impact		Directive—UE 2017/745 Tittle 21 FDA Federal Code	Develop biomaterials with active antimicrobial surfaces, combined with antibiotics, in accordance with safety regulations for human health
**Bhatt et al.** 2023 **[15]**	Assessment of nanobiological factors that influence the antimicrobial effects	Morphology of graphene nanosheets Nano-wrapping	Oxidative stress Photothermal irradiation Electrical conductivity	Correlation considered	GO rGO	Various Gram + and Gram − bacteria	Observed (improved by different graphene’s applications)	Coated graphene has lower cytotoxicity		Need for in vivo testing	Functionalizing biomaterials with specific antimicrobial properties
**Lazar et al.** 2023 **[34]**	Review of the recent progress of using graphene-related materials in biomedical applications		Oxidative stress Photothermal irradiation Electrical conductivity		GO (0.25 wt%) rGO (Al_2_O_3_)		Need for in vivo testing	Conflicting results	Tissue regeneration is enhanced by the diverse applications of graphene	Potentially hazardous depending on the dose and type of graphene.	Need for a comprehensive assessment of the toxic potential of these materials for human health
**Williams et al.** 2023 **[12]**	Evaluation of in vitro and in vivo studies on cytotoxicity	Morphology of graphene nanosheets Nano-wrapping	Oxidative stress Electrical conductivity	Correlation considered	GO associated with copper or silica rGO (1 µg/mL—demonstrates toxicity)		Need for in vitro and in vivo studies	Present due to cellular interaction with graphene	Increased stem cell differentiation due to high surface energy of graphene	Penetration of graphene into the nucleus of human cells Genotoxicity	Graphene, in combination with other nanoparticles, enhances the proliferation and differentiation of stem cells by improving biocompatibility
**Kumar et al.** 2023 **[35]**	Evaluate the biofilm resistance and antibacterial properties of sulfonated PEEK conjugated with GO and nisin	Morphology of graphene nanosheets	Oxidative stress Pore formation Lipid trapping		GO GO + Nisin	*S. aureus*	Observed				Conjugation of sulfonated PEEK with GO and nisin resulted in synergistic bactericidal efficacy, along with reduced bacterial adhesion and biofilm formation
**Ferreira et al.** 2024 **[32]**	Antimicrobial effectiveness of graphene incorporated into PMMA	Morphology of graphene nanosheets Nano-wrapping	Oxidative stress		GO		Observed (lower graphene doping in the biomaterial improves biocompatibility)	Lower graphene doping in biomaterial results in a greater effect			Studies show high antimicrobial effect with low doses graphene
**Singh et al.** 2025 **[17]**	Investigate the effects of incorporating graphene derivatives into polymer materials	Morphology of graphene nanosheets Nano-wrapping	Oxidative stress Electrical conductivity		GO rGO		Observed (better cellular response)	Need for in vivo testing	Effective bone integration in graphene-doped dental implants		Doped polymeric surfaces present high antimicrobial effect
**Huang et al.** 2024 **[36]**	Characterize the mechanical properties, antimicrobial effect and bioactivity of PEEK, sulfonated PEEK, and GO-grafted sulfonated PEEK	Morphology of graphene nanosheets	Bacterial phospholipid translocation		GO	*E. coli* *S. aureus*	Cytocompatibility observed in all samples (enhanced cell proliferation in GO-sulfonated PEEK)		GO-grafted sulfonated PEEK enhanced adhesion and osteogenic activity of a mouse osteoblastic cell line		GO-grafted sulfonated PEEK exhibited enhanced bactericidal activity and biomineralization capacity compared to unmodified PEEK, while maintaining similar mechanical properties

* The antimicrobial activity was quantitatively measured using disk diffusion assays, broth dilution methods, and live/dead viability assays. In some studies, graphene-based surfaces themselves served as the culture substrate for antimicrobial testing. ^#^ When not used as composites, graphene oxide (GO) and reduced graphene oxide (rGO) were applied as aqueous suspensions, whereas graphene nanoplatelets (GNP) were used in powder form. ^‡^ Testing methods were performed following ISO standards: PMMA—Polymethylmethacrylate; PEEK—Polyetheretherketone.

## 4. Discussion

### 4.1. Broad-Spectrum Antimicrobial Activity

The antimicrobial effect of different types of graphene is not always achieved at low concentrations, unlike the enhancement of the mechanical properties of biomaterials. Determining the optimal graphene concentration by weight for material functionalization remains a significant challenge, as it directly influences antimicrobial effectiveness, cytotoxicity, biocompatibility [8,9,12,13,24,25,27,28,33,37], and the impact on mechanical properties [16,19,26,32], whether positive or negative [9,38].

As thoroughly reviewed by Bhatt et al. (2023) and Zhou et al. (2022), the chemical, surface, and morphological properties of graphene are closely associated with its antipathogenic potential [15,19]. Longer and thinner functionalized graphene nanosheets exhibit greater antimicrobial effect [7,13,16]. Accordingly, increasing the graphene concentration in a biomaterial enhances the surface area [34] and interactions at graphene–bacteria interfaces, thereby improving antimicrobial effectiveness [8,9,13,16,28,32,33].

However, high concentrations of graphene induce the “stacking” of sheets on the material’s surface, imparting a high antimicrobial potential while reducing mechanical properties [12,13,16,19,28,33,37]. In fact, low concentrations of graphene exhibit excellent mechanical properties but minimal bactericidal effects [16,19,28]. To achieve a more comprehensive antimicrobial effect, higher concentrations of incorporated graphene are required, particularly for the rGO variant [10]. In contrast, at low concentrations, GO functionalized into dental biomaterials enhances broad-spectrum antimicrobial effectiveness while maintaining optimal mechanical properties [9,11,16,24,25,27,28,32,34,38].

In vitro studies demonstrate the excellent mechano-bactericidal activity of graphene when present on the surface of functionalized materials [12,13,27]. This effect occurs because the nanosheets physically disrupt bacterial and fungal membranes, leading to lysis [7,8,9,10,11,13,25,26,27,32,33]. Another proposed physical mechanism involves the nano-wrapping of microorganisms within the atomic defects of graphene nanosheets, trapping them in its two-dimensional mesh. Combined with glucose restriction in the medium, this entrapment ultimately leads to cell death [32].

Besides the aforementioned mechanical mechanisms, additional physical and chemical pathways have been attributed to the antimicrobial activity of graphene. In fact, the chemical properties of graphene at the molecular level of interface exposure are considered highly effective in exerting antimicrobial effects, particularly through mechanisms involving oxidative stress, photothermal irradiation, and surface electrical conductivity [8,9,27]. Specifically, the surface oxidation of graphene exhibits high electrical conductivity and may induce bacterial cell death through electrostatic interactions (e.g., electrical shock) [3,12,14,28]. It is hypothesized that graphene’s extremely low density, approximately 0.0595±g/cm^3^ per sheet [39], may also contribute to its antimicrobial mechanisms [14]. The shape, size, concentration, and morphological type of graphene nanosheets are directly associated with the antimicrobial outcome [7,12,13,16,28,32]. The intracellular biochemical processes of bacteria in the presence of functionalized biomaterials should be explored, as they may represent a novel pathway underlying the diverse antimicrobial effects observed in graphene studies.

The antimicrobial applications of graphene–polymer composites in nanomedicine have been extensively reviewed elsewhere [15]. In this context, the functionalization of dental materials with graphene, combined with other carbon derivatives such as carbon nanotubes and fullerenes, has been the subject of increasing research [12,13,16]. Both GO and carbon nanotubes have exhibited promising antimicrobial activity against antibiotic-resistant bacterial infections [12,13,16,28,33], with graphene presenting broad-spectrum antimicrobial effects comparable to antibiotics such as streptomycin and kanamycin [31]. Furthermore, functionalization with graphene and other carbon derivatives has shown favorable outcomes in terms of biocompatibility [13,28,32]. Avcu and colleagues critically reviewed the incorporation of graphene-related materials into biodegradable polymer matrices such as chitosan [18]. This combination enables the development of high-performance polymer composites with enhanced antibacterial activity, drug-loading capacity, and superior mechanical properties compared to the original polymer matrix [18]. In the field of dental medicine, the incorporation of graphene into the synthetic polymer PMMA has also demonstrated significant antimicrobial effectiveness, limiting the adhesion and growth of microorganisms on the resin surface [26,32]. Moreover, the use of GO as a coating for sulfonated PEEK—a thermoplastic polymer—has shown improved antimicrobial properties, enhanced biomineralization capacity, and superior mechanical properties [35,36], making it a promising strategy for dental and orthopedic implants.

The critical issue that remains unresolved is the extent of graphene’s antimicrobial effect, as no in vivo studies involving its use in humans have been conducted to date [16]. The effects observed at the interfaces are supported by laboratory studies, which demonstrate the eradication of bacteria and fungi within a short exposure period. However, asserting that graphene exhibits a significant antimicrobial effect in dental medicine remains clinically unsupported [26,28]. Furthermore, beyond the exposure duration to graphene, future studies should investigate parameters such as the total lethality rate and the initial composition of the oral microbiome, given its variability among individuals [12,13,28].

### 4.2. Biological Responses to Graphene

The biocompatibility of graphene and its derivatives has raised some concerns [12,33]. Although GO has exhibited the best biocompatibility with living tissues [26], some in vivo studies in mice have reported negative outcomes, including cellular apoptosis, hemolysis, tissue inflammation in the liver and nervous system, and DNA damage in bone marrow cells [13,26].

As mentioned above, high doses of graphene incorporation enhance antimicrobial activity [16,28], but the cytotoxicity may increase as well [9,12,13,28]. Achieving optimal tissue biocompatibility is essential for effective tissue repair and regeneration [17,18].

The increasing incidence of infectious diseases, including those caused by antibiotic-resistant oral bacteria, represents a major threat to human health, particularly in elderly populations. Functionalized graphene-based nanomaterials have demonstrated the potential to inhibit the colonization of these microorganisms and may be associated with a decreased risk of systemic diseases [19,32,34]. The review by Butler et al. (2023) also acknowledges the major clinical challenges posed by the continuous emergence of antimicrobial-resistant microorganisms and discusses international efforts to develop alternative antimicrobial agents [13]. The immobilization of GNP and GO as nanocoatings to protect surfaces from pathogen colonization represents a significant area of research in dental and maxillofacial implants. Such strategies enable localized, rather than systemic, antimicrobial effects, thereby reducing the adverse side effects of antibiotics and decreasing the likelihood of selecting for antibiotic-resistant bacteria [13].

While these findings support the promising potential of graphene-based materials in combating infections, they also raise critical safety considerations. Specifically, high antimicrobial potency raises concerns, as it may disrupt oral ecosystem homeostasis by eliminating both pathogenic and beneficial microorganisms [16]. Currently, no studies have specifically addressed the impact of graphene functionalization on beneficial microorganisms, highlighting a critical gap in the research. Therefore, determining the appropriate dosage of graphene in functionalized biomaterials—while balancing cytotoxicity, biocompatibility, and antimicrobial efficacy—remains a significant challenge.

Despite extensive research on graphene, discrepancies in its use persist, including the lack of a standardized protocol and the control of surface properties across different production techniques [13,19,26]. Graphene and its derivatives are often used in uncontaminated and defect-free samples, but unpredictable defects can arise during graphene synthesis, altering its electronic structure, sensitivity, and reactivity. These changes have a direct impact on its bactericidal potential [24,27].

Occupational exposure to engineered nanomaterials, including graphene-based materials, is a particular concern in dentistry, as respiratory exposure may occur during drilling procedures or during the fabrication and adjustment of dental prostheses, which generate aerosols and ultrafine particles, respectively [13]. While the WHO has published general principles for handling nanomaterials (e.g., WHO Guidelines on Protecting Workers from Potential Risks of Manufactured Nanomaterials, 2017), dentistry-specific safety guidelines for the use of graphene and its derivatives are still lacking. This gap highlights the need for further toxicological and clinical studies to establish safety standards that account for nanoscale properties, as well as clear guidelines for managing occupational exposure risks associated with these materials [13,24,32].

### 4.3. Future Perspectives

Despite the extensive research on the antimicrobial, biocompatibility, and cytotoxic properties of graphene-based materials, there remains a lack of standardized testing protocols across the literature. The most commonly used methods to quantitatively assess antimicrobial activity include disk diffusion or broth dilution methods; however, some studies do not adhere to internationally validated standards. Similarly, biocompatibility assessments are often performed without explicitly following ISO guidelines—particularly ISO 10993 [40], which is critical for the biological evaluation of medical devices. Such methodological inconsistencies limit the inclusion of certain studies in systematic/scoping reviews and hinder cross-study comparisons, ultimately impairing the clinical translation of laboratory findings.

Future research should aim to determine the optimal concentration and type of graphene for incorporation into or surface coating of dental biomaterials, tailored to target specific bacterial or fungal species [26]. This is crucial due to the varying mechanisms of cell death induced by exposure to graphene surfaces. Optimizing these parameters will enhance biocompatibility, reduce cytotoxicity, and improve the overall antimicrobial potential [27]. To validate the antimicrobial efficacy and safety of graphene for therapeutic use in humans, further in vitro and in vivo studies are needed. These investigations should follow standardized health protocols, focusing on biocompatibility, cytotoxicity, biological benefits, potential risks, graphene concentrations, and effective clinical applications in dental medicine [9,12,13,19,27,34]. Additionally, the combination of graphene with other nanoparticles and functional groups, as well as its dispersibility, hydrophilicity, and solubility, presents significant scientific interest due to the promising antimicrobial effects observed [12,16,32].

## 5. Conclusions

- Graphene functionalization of dental biomaterials offers a significant advancement in modulating the oral ecosystem and provides a valuable strategy for combating traditional drug-resistant microorganisms.
- In the field of dental medicine, the incorporation of graphene into natural biodegradable polymer matrices (e.g., chitosan) or synthetic polymers (e.g., PMMA, PEEK) represents a promising approach not only for enhancing antimicrobial activity but also for supporting osteogenic and tissue regenerative applications.
- The optimal concentration of graphene for functionalization of dental biomaterials, aiming to achieve antimicrobial efficacy without compromising biocompatibility, remains under investigation. This includes the need for standardized production protocols, defined clinical applications in dental medicine, and regulatory guidelines from the WHO regarding handling procedures and occupational safety.
- Future research directions should include optimal dose–response studies, standardized cell-based toxicity assays, long-term in vivo investigations (to evaluate the effects of chronic exposure), studies on the modulation of oral microbiome (to assess how the balance of beneficial vs. pathogenic microorganisms is affected), and occupational exposure assessments.

## Figures and Tables

**Table 1:** Description of PICO strategy.

Acronym	Definition	Description
P	Problem	Understand the antimicrobial effect of various types of graphene functionalized into dental biomaterials.
I	Intervention	Incorporation of graphene (in various forms and concentrations) into dental biomaterials for antimicrobial purposes.
C	Comparison	Compare the antimicrobial effect among various types of graphene and with other antimicrobial agents (antibiotics).
O	Outcome	Increasing the antimicrobial effect of the biomaterials functionalized with graphene.

**Table 2:** Inclusion and exclusion criteria.

Inclusion Criteria	Exclusion Criteria
In vivo and in vitro studies Narrative and systematic reviews Studies published until 31 December 2024 Studies written in English Studies addressing the antimicrobial effect of various types of graphene functionalized in dental biomaterials	Prospective and retrospective studies Opinion articles Articles with unavailable text Articles on graphene functionalized in non-dental biomaterials

## Data Availability

No new data were created or analyzed in this study. Data sharing is not applicable to this article.

## Abbreviations

The following abbreviations are used in this manuscript:

GNP	Graphene nanoplatelets
GO	Graphene oxide
PEEK	Polyetheretherketone
PMMA	Polymethylmethacrylate
rGO	Reduced graphene oxide
WHO	World Health Organization
